# Autosomal Recessive *RDH12*-Leber Congenital Amaurosis Retinal Organoids Suggest Phospholipid Pathway Dysregulation

**DOI:** 10.3390/ijms27198880

**Published:** 2026-10-06

**Authors:** Cécile Méjécase, Lyes Toualbi, Ya Zhou, Riccardo Cheloni, Neelima Nair, Nicholas Owen, Hajrah Sarkar, Jieren Bornia, Maria Toms, Pablo Soro-Barrio, Mariya Moosajee

**Affiliations:** 1 Development, Ageing and Disease, UCL Institute of Ophthalmology, London EC1V 9EL, UK; c.mejecase@ucl.ac.uk (C.M.);; 2 Ocular Genomics and Therapeutics, The Francis Crick Institute, London NW1 1AT, UK; 3 Electron Microscopy Science Technology Platform, The Francis Crick Institute, London NW1 1AT, UK; 4 Department of Genetics, Moorfields Eye Hospital NHS Foundation Trust, London EC1V 2PD, UK; 5 Bioinformatics and Biostatistics Science Technology Platform, The Francis Crick Institute, London NW1 1AT, UK

**Keywords:** retinal organoids, RDH12, autosomal recessive, Leber congenital amaurosis, induced pluripotent stem cells, phospholipid transport

## Abstract

NADPH-dependent retinol dehydrogenase 12 (RDH12) reduces all-*trans*-retinal in photoreceptor inner segments to regulate the phototransduction cascade and control the concentration of retinoids in the retina. Variants in *RDH12* have been reported to cause an array of retinal disease phenotypes, including autosomal recessive (AR) Leber congenital amaurosis (LCA), a severe form of childhood blindness, and autosomal dominant (AD) retinitis pigmentosa (RP), a milder late-onset rod-dominant disease. Animal models have failed to recapitulate the clinical phenotype; hence, we generated human induced pluripotent stem cell (hiPSC) derived retinal organoids (RO) from a patient with homozygous missense variant c.619A>G p.(Asn207Asp) in *RDH12* (*RDH12*-AR), a patient with heterozygous c.759del p.(Phe254Leufs*24) (*RDH12*-AD), and an unaffected control. *RDH12*-AR ROs develop shortened rod and cone photoreceptors (from the outer limiting membrane to the tip of the outer segment), which lack RDH12 localisation in the inner segment. Bulk transcriptomic analysis revealed cone marker and apoptosis dysregulation in *RDH12*-AR ROs compared to unaffected controls, but phospholipid transport is perturbed in *RDH12*-AR ROs compared to *RDH12*-AD. Our study helps to decipher the distinct disease pathways involved in *RDH12* retinopathies; however, further modelling from additional patients with isogenic controls will strengthen the findings and aid identification of targets for therapeutic approaches.

## 1. Introduction

Retinoids are a class of vitamin A derivatives that are highly regulated in the visual cycle within the photoreceptors, retinal pigment epithelium (RPE), and Müller glia of the retina [1]. Photoactivation of rhodopsin and opsin releases all-*trans*-retinal in photoreceptor outer segments; the majority is reduced to all-*trans*-retinol by retinol dehydrogenase 8 (RDH8), but some leaks into the inner segments for reduction by the NADPH-dependent retinol dehydrogenase 12 (RDH12) [2]. All-*trans*-retinol is then transported into the RPE for recycling into all-*cis*-retinal to enter into a new cycle. In parallel, all-*trans*-retinal molecules can interact with phosphatidylethanolamine (PE) in photoreceptor disc membranes to create *N*-retinylidene-PE [3], which is transported by the ATP-binding cassette (ABC) transporter, subfamily A, member 4 (ABCA4) in the photoreceptor cytoplasm. *N*-retinylidene-PE is then hydrolysed to separate PE and all-*trans*-retinal for re-entry into the visual cycle. In not fully defined conditions, *N*-retinylidene-PE can interact with a second all-*trans*-retinal molecule to form fluorescent bisretinoids, such as phosphatidyl-pyridinium bisretinoid (A2PE), a precursor of lipofuscin [3].

Disruption of genes involved in the regulation of the retinoid cycle and bisretinoid formation leads to inherited retinal diseases (IRDs) displaying both genetic and clinical heterogeneity [1,3,4]; for example, pathogenic variants in *RDH12* can cause (i) autosomal recessive Leber congenital amaurosis (LCA)/early-onset severe retinal dystrophy (EOSRD), retinitis pigmentosa (RP), cone-rod dystrophy (CRD), and macular dystrophy [2,5,6], as well as (ii) a rare autosomal dominant RP [7,8,9]. Autosomal recessive *RDH12*-related LCA is characterised by progressive macular degeneration with disorganised retinal layers associated with early-onset reduced scotopic and photopic electroretinogram (ERG), leading to certifiable blindness before the third decade [10]. Autosomal dominant *RDH12*-RP involves later onset nyctalopia and progressive visual field loss, with preserved central vision into late adulthood. Adaptive optics scanning laser ophthalmoscopy (AOSLO) revealed cone density was slightly reduced at the fovea, but this was significantly reduced at eccentric distances from the centre [7,8,9].

In vitro cell models overexpressing *RDH12* in HEK-293 stable lines have been developed and reported; all-*trans*-retinal induces oxidative stress and cell death in *RDH12* mutant cells [11]. Further mouse and zebrafish models with homozygous *Rdh12* variants failed to recapitulate early-onset retinal phenotypes observed in patients [11,12]. We previously reported a retinal organoid (RO) model derived from human induced pluripotent stem cells (hiPSC) obtained from a patient with *RDH12* autosomal dominant RP (*RDH12*-AD), which recapitulated the phenotype observed in the patient [13]: *RDH12*-AD photoreceptors were less abundant and shorter than control photoreceptors, and cone function was altered. No RO models of autosomal recessive *RDH12* LCA (*RDH12*-AR) have been previously reported. Hence, in this study, we employed hiPSC technology to generate ROs derived from a 40-year-old female affected with *RDH12*-AR [14,15], and performed transcriptomic analysis between unaffected control and *RDH12*-AD models to provide insights into disease pathophysiology.

## 2. Results

### 2.1. RDH12-AR Retinal Organoids Develop Photoreceptors That Are Deficient in RDH12 Protein

**Figure 1:**
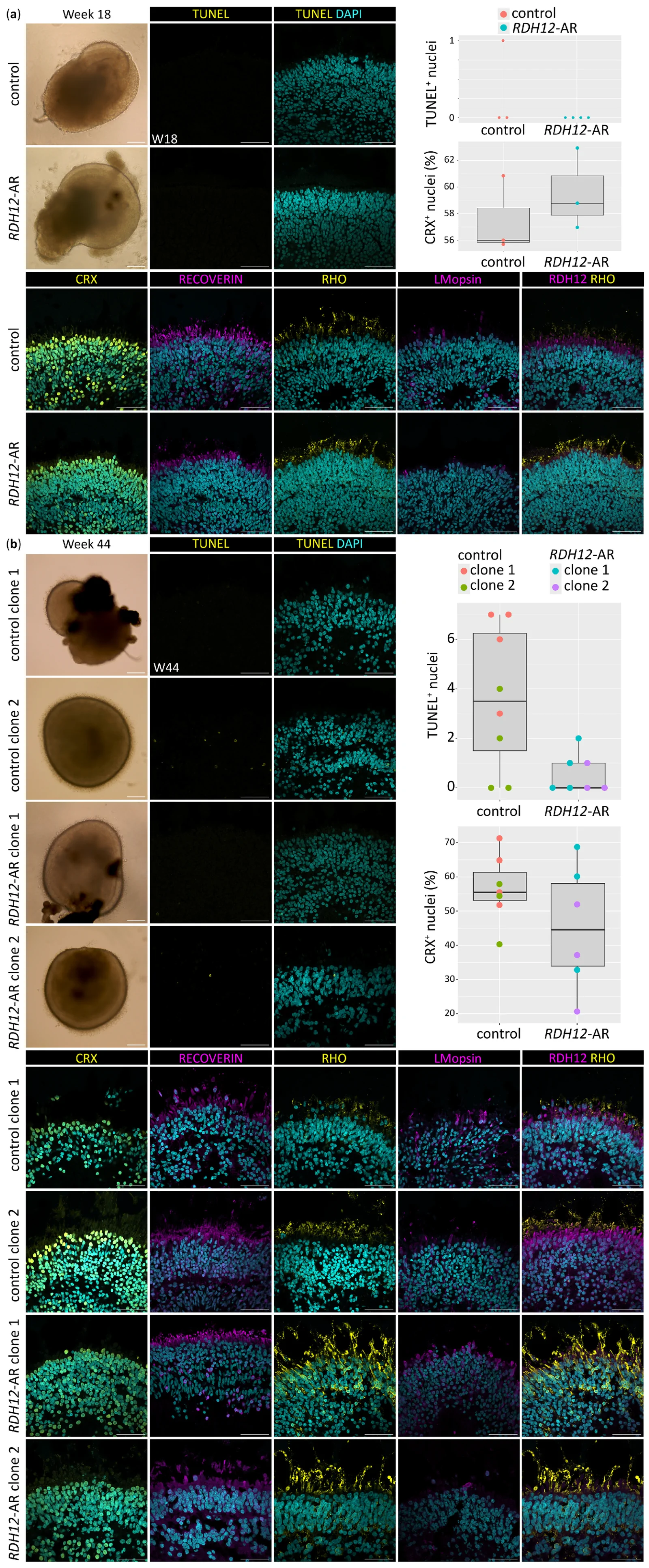
Both unaffected control and *RDH12*-AR retinal organoids developed both rod and cone photoreceptors but RDH12 was absent from photoreceptor segments in *RDH12*-AR. (**a**) *RDH12*-AR retinal organoids developed rod and cone photoreceptors at week 18, with absence of RDH12 in photoreceptor segments. *n* = 3 per line, from 2 differentiation batches, from 2 clones. TUNEL assay analysis (*t*-test, *p* = 0.42) and CRX-positive cell quantification (*t*-test, *p* = 0.45) reveal similar cell viability and similar numbers of photoreceptor cells in the *RDH12*-AR model compared to control retinal organoids. (**b**) RDH12 was absent from photoreceptor inner segments in mature *RDH12*-AR retinal organoids at week 44. *RDH12-AR* cells survived significantly more than control cells (*p* = 0.02) in mature retinal organoids, while the number of photoreceptor cells (*p* = 0.21) remained unchanged. *n* = 3 per line, from 2 differentiation batches, from 2 clones. CRX, RECOVERIN: photoreceptor precursors; RHO: rod; L/M-opsin: cone. Scale bar: 200 µm for brightfield images; 50 µm.

ROs were characterised at week 18 and 44 of differentiation (Figure 1). At week 18, both unaffected control and *RDH12*-AR developed mature laminated ROs with rod and cone photoreceptors, and no significant difference in cell viability (*p* = 0.42) or in CRX-positive nuclei (*p* = 0.45; Figure 1a). RDH12 protein was absent from photoreceptor segments. At week 44, *RDH12*-AR retinal organoids (lacking any RDH12 expression) showed significantly less cell death (*p* = 0.02), but the number of photoreceptors remained similar to control retinal organoids (*p* = 0.21) (Figure 1b). RNA in situ hybridisation shows similar *RDH12* mRNA levels in both control and *RDH12*-AR retinal organoids (*p* = 0.083, Figure S1).

### 2.2. Photoreceptors Are Shortened in RDH12-AR Retinal Organoids Compared to Control Retinal Organoids

**Figure 2:**
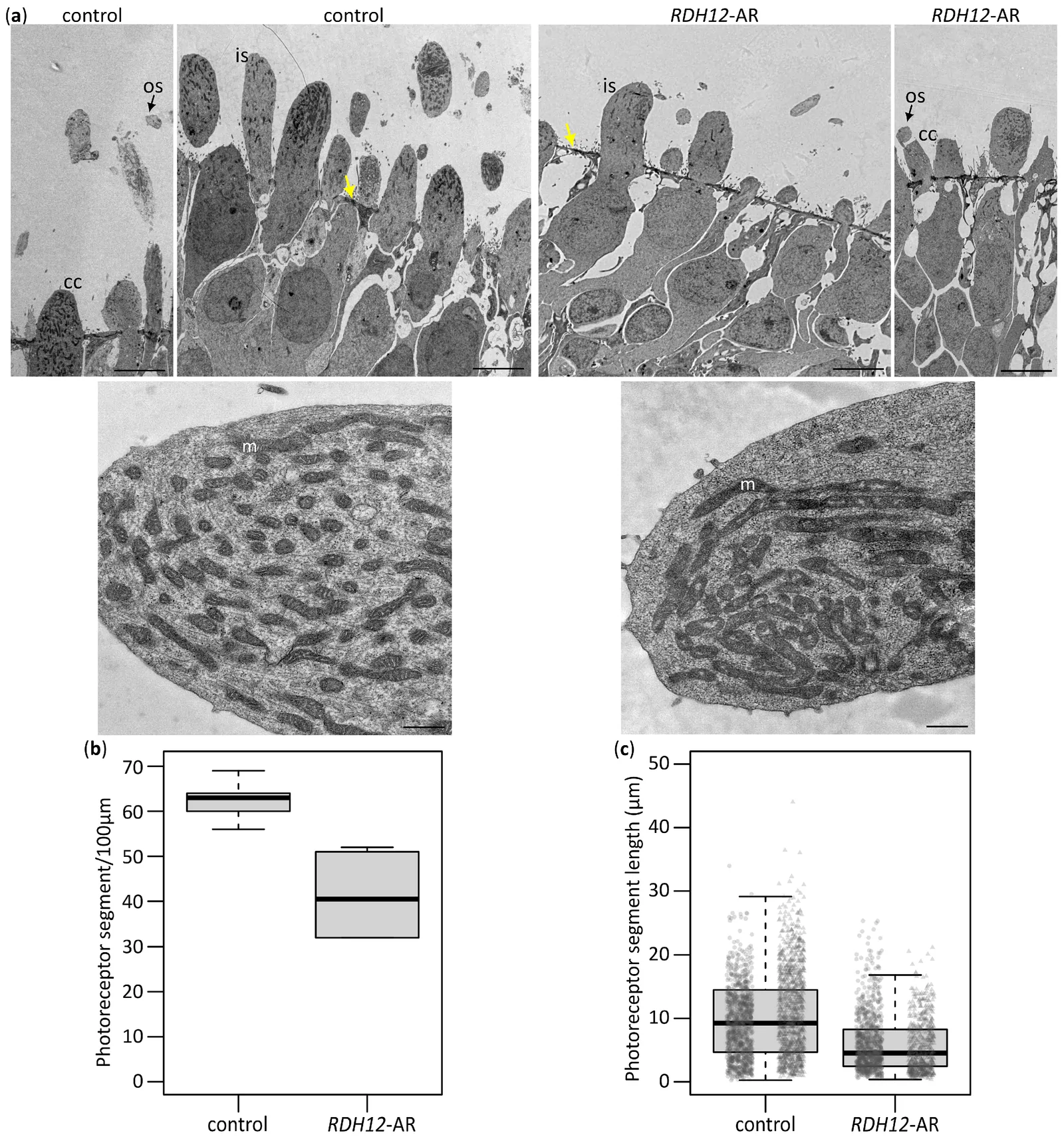
*RDH12*-AR photoreceptors are shorter than control photoreceptors at week 37. (**a**) Photoreceptor ultrastructure is similar in both unaffected control and *RDH12*-AR mature organoids, with developing inner segment (is) containing mitochondria (m), presumptive outer segments (os), and connecting cilium (cc). The outer limiting membrane (OLM) is indicated with yellow arrows. Scale bar: 5 µm (top), 500 nm (bottom). (**b**) The number of photoreceptors (mean ± SEM) per 100 µm in *RDH12*-AR and control retinal organoids was similar. (**c**) *RDH12*-AR photoreceptor segments, from the outer limiting membrane to the tip of the presumptive outer segment, are shorter than in unaffected controls. Three regions from 2 retinal organoids per line, from 1 differentiation batch, were studied: a total of 2097 and 1535 photoreceptor segments, from the outer limiting membrane to the tip end, were counted and measured in control and *RDH12*-AR samples, respectively; statistical analysis cannot be performed as only two retinal organoids per group were analysed.

Ultrastructure analysis of two retinal organoids at week 37 revealed photoreceptors with a developing outer segment (os), an inner segment (is) with mitochondria (m), and an intact outer limiting membrane (OLM) in both unaffected control and *RDH12*-AR retinal organoids (Figure 2a). Photoreceptor density was comparable between the two models: unaffected control and *RDH12*-AR organoids developed 62 ± 5 photoreceptors and 41 ± 10 photoreceptors per 100 µm, respectively (Figure 2b). However, *RDH12*-AR photoreceptor segments, from the outer limiting membrane to the tip of the presumptive outer segment, were shorter (5.89 ± 4.44 µm, range between 0.38 µm and 25.34 µm) than control (10.09 ± 6.48 µm, range between 0.25 µm and 44.02 µm) (Figure 2c).

### 2.3. RDH12-AR, RDH12-AD and Unaffected Retinal Organoids Appear Resistant to All-Trans-Retinal-Induced Stress

**Figure 3:**
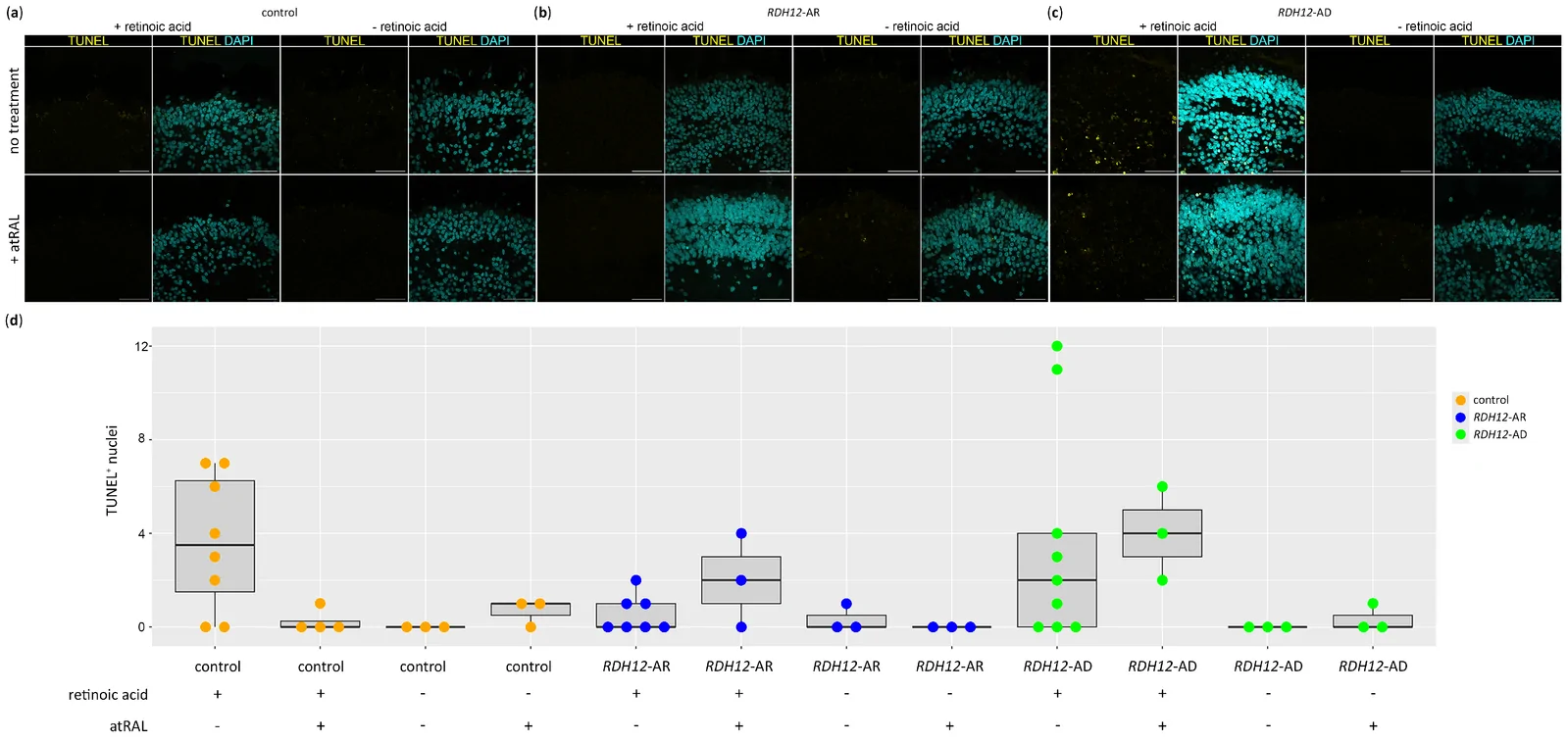
Control, *RDH12*-AR and *RDH12*-AD mature retinal organoids at week 41–44 appear resistant to stress induced by all-*trans*-retinal (atRAL). Mature retinal organoids were cultivated in medium with or without retinoic acid (no RA) for 24 h, then further stressed with all-*trans*-retinal (atRAL) for 24 h (*n* = 3–8 per condition). (**a**) Control, (**b**) *RDH12*-AR and (**c**) *RDH12*-AD retinal organoids did not show a detectable response to stress induced by all-*trans*-retinal (atRAL). Apoptosis was studied with TUNEL assay. Scale bar: 50 µm. (**d**) No significant difference in apoptosis was noted between any retinal organoid models (*p* > 0.99, two-way ANOVA).

A previous study reported all-*trans*-retinal induces oxidative and endoplasmic reticulum (ER) stress in HEK-293 cells overexpressing mutant *RDH12* [11]. Unaffected control, *RDH12*-AR and *RDH12*-AD retinal organoids at week 44 were stressed for 24 h with 100 µM concentration of all-*trans*-retinal (Figure 3 and Figure S2). As the continuous presence of retinoic acid in the medium from day 84 could protect against all-*trans*-retinal-induced stress [16,17], further week 41 retinal organoids were cultivated for 24 h without retinoic acid and then treated during the following 24 h with all-*trans*-retinal (Figure 3 and Figure S2). Both control, *RDH12*-AR and *RDH12*-AD retinal organoids appeared resistant to all-*trans*-retinal induced cell death (TUNEL assay, *p* > 0.99 for all groups), oxidative (8-OHdG, Acrolein) and ER (CHOP/DDIT3, BiP/HSPA5) stress and induced autophagy (LC3), in the absence or presence of retinoic acid in the medium. Hydrogen peroxide (H_2_O_2_) was used as a positive control in control organoids, but control retinal organoids did not show a detectable stress response through cell death (TUNEL assay, *p* > 0.86), oxidative (8-OHdG, Acrolein) and ER (CHOP/DDIT3, BiP/HSPA5) stress and induced autophagy (LC3), in the absence or presence of retinoic acid in the medium (Figure S2A).

### 2.4. RDH12-AR Retinal Organoids Reveal a Transcriptomic Signature Distinct from Unaffected Control and RDH12-AD Mature Organoids

**Figure 4:**
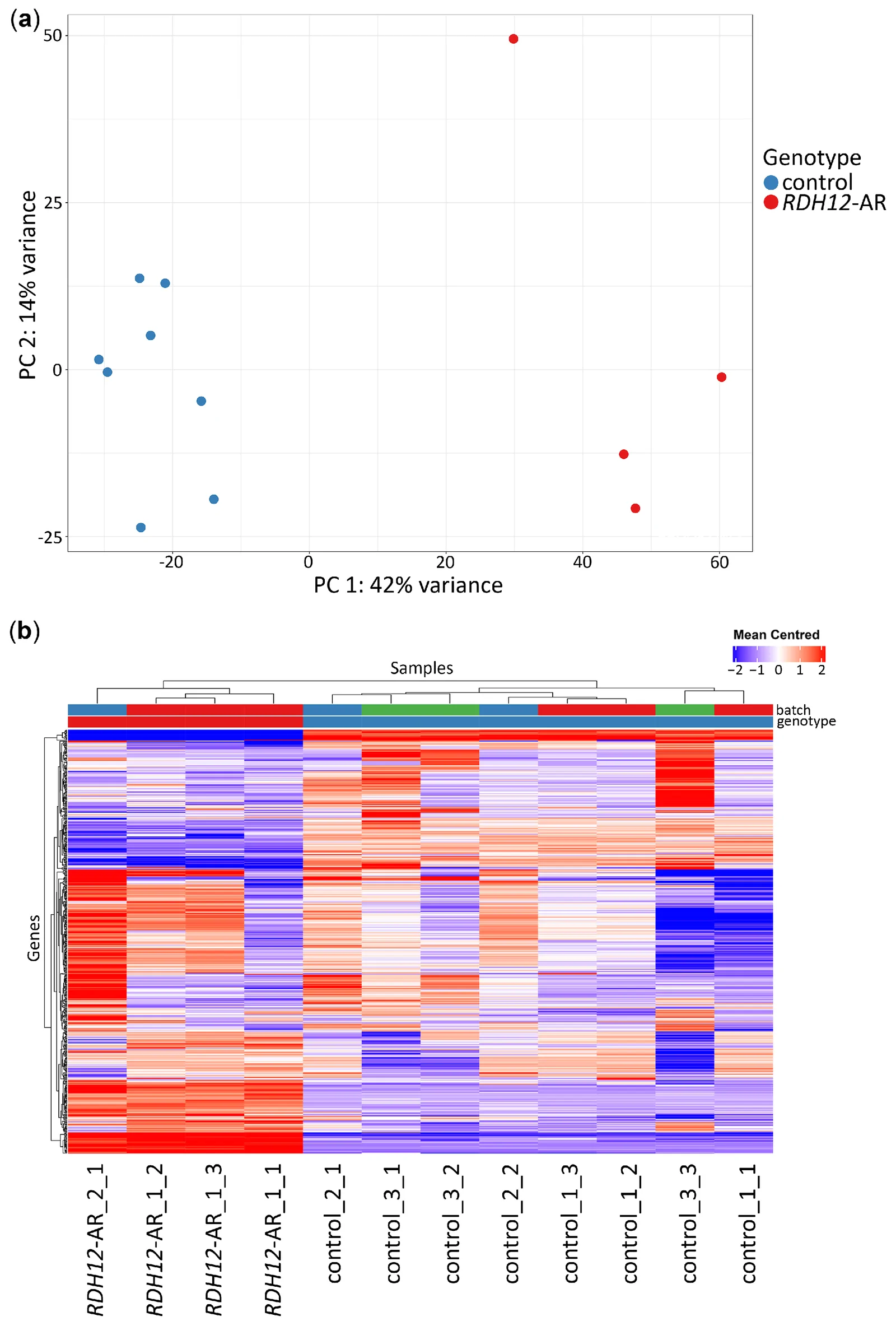
*RDH12*-AR retinal organoids differentially express more than 3000 genes compared to unaffected control mature organoids at week 44. (**a**) Principal component analysis separated control and *RDH12*-AR samples in two groups, with PC1 displaying 42% variance. (**b**) A general heatmap depicted differential gene expression in *RDH12*-AR (red in genotype line) and in control samples (blue in genotype line). Standard deviation around the mean (mean-centred) highlights upregulated genes in red and downregulated genes in blue.

Disease-mechanisms associated with human *RDH12*-related LCA remain unclear. To unravel the pathophysiology, transcriptomic analysis was performed in mature day 303 (week 44) *RDH12*-AR and unaffected control retinal organoids. Each group is distinct on the principal component (PC) analysis plot with PC1 variance reaching 42% (Figure 4a). *RDH12*-AR retinal organoids differentially expressed 3011 genes, compared to unaffected mature organoids, with 1520 downregulated and 1491 upregulated (Figure 4b). Gene ontology (GO) over-representation highlighted nucleoside-triphosphatase regulator activity (including GO:0060589, GO:0005096, GO:0030695), extracellular matrix structural constituent (GO:0005201), growth factor binding (GO:0019838) annotations in *RDH12*-AR (Table S1), where most of the genes were upregulated.

**Figure 5:**
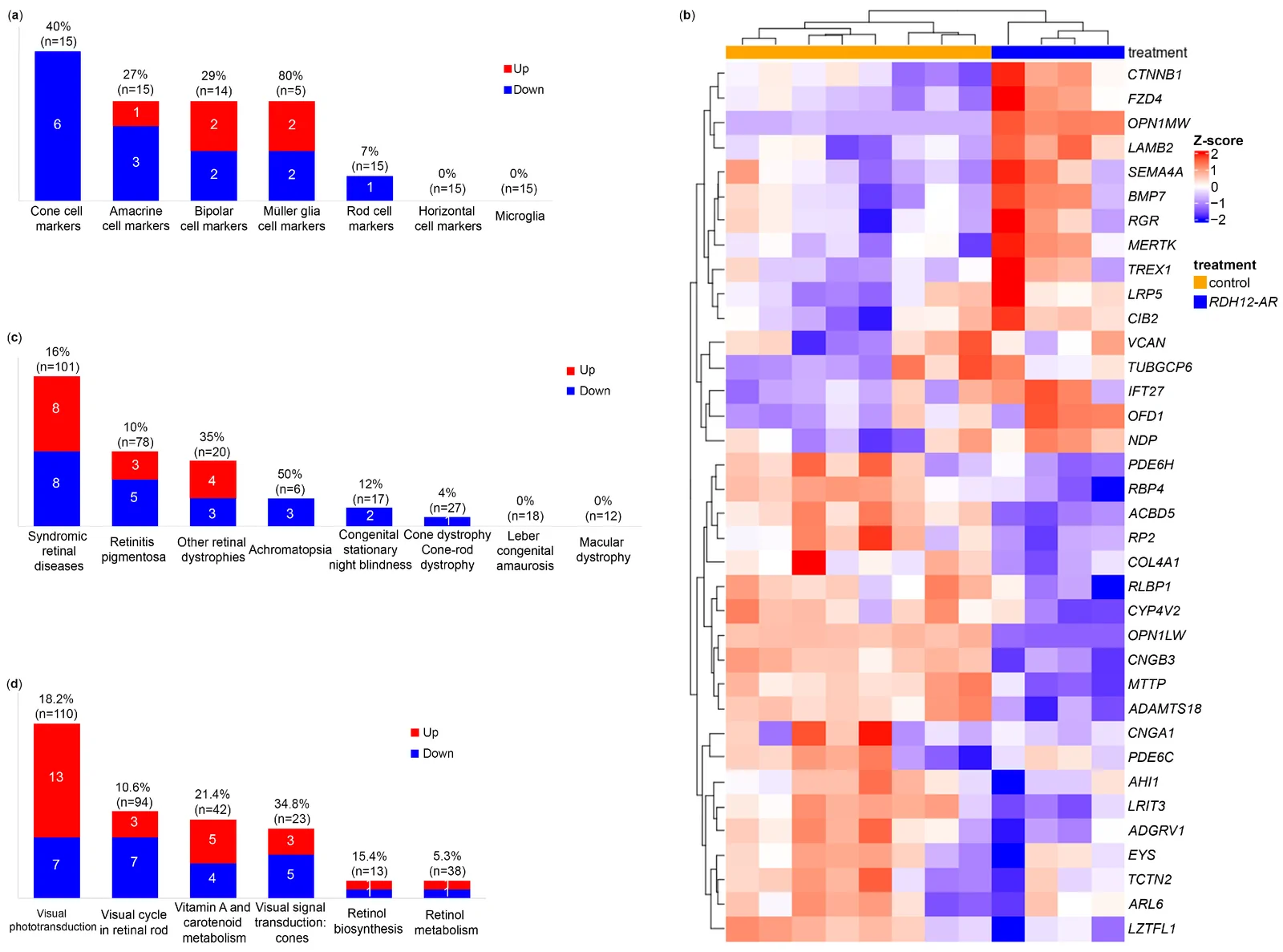
*RDH12*-AR retinal organoids differ from unaffected controls at week 44 with dysregulation of cone cell markers, genes associated with retinal dystrophies and genes involved in RDH12 pathways. (**a**) Cone photoreceptor cells are downregulated in *RDH12*-AR mature organoids. (**b**) Thirty-six genes associated with retinal dystrophies were differentially expressed, with 20 downregulated and 16 upregulated. Standard deviation around the mean (z-score) highlights upregulated genes in red and downregulated genes in blue. (**c**) Thirty-six genes associated with retinal dystrophies are significantly differentially expressed in mature *RDH12*-AR retinal organoids. (**d**) Thirty-five genes involved in RDH12 pathways are differentially expressed in mature *RDH12*-AR retinal organoids. *n* indicates the number of genes per group.

Downregulation of cone photoreceptor cell markers was seen in *RDH12*-AR mature organoids [18] (Figure 5 and Table S2). Interestingly, three pro-apoptotic markers are downregulated in *RDH12*-AR organoids: *TP53AIP1* (LCF −1.586; *padj* < 1.008 × 10^−2^), *CASP3* (LCF −0.622; *padj* < 8.051 × 10^−6^) and *MOAP1* (LCF −0.530; *padj* < 1.251 × 10^−4^). We investigated the expression patterns of genes associated with genetic eye disease in *RDH12*-AR mature organoids. Thirty-six genes associated with retinal dystrophies were differentially expressed, with 20 downregulated and 16 upregulated (Figure 5b,c and Table S3). These genes were previously reported to be associated with syndromic retinal diseases (*ACBD5*, *ADAMTS18*, *ADGRV1*, *AHI1*, *BMP7*, *COL4A1*, *CTNNB1*, *CYP4V2*, *IFT27*, *LAMB2*, *LZTFL1*, *NDP*, *OFD1*, *TCTN2*, *VCAN*, *TUBGCP6*), retinitis pigmentosa (*ADAMTS18*, *ARL6*, *EYS*, *MERTK*, *OFD1*, *RLBP1*, *RP2*, *SEMA4A*), other retinal dystrophies (*FZD4*, *LRP5*, *MTTP*, *NDP*, *OPN1LW*, *OPN1MW*, *RBP4*), achromatopsia (*CNGB3*, *PDE6C*, *PDE6H*), congenital stationary night blindness (*LRIT3* and *RLBP1*) or cone dystrophy/cone-rod dystrophy (*PDE6C*). *RLBP1* downregulation in *RDH12*-AR mature retinal organoids is confirmed by RNA in situ hybridisation (*p* = 0.018, Figure S3A). A trend toward reduction was observed with *RBP4* mRNA (*p* = 0.32, Figure S3B).

RDH12 was previously reported to be associated with phototransduction, vitamin A metabolism, retinol biosynthesis and metabolism [2]. Photoreceptor function is highly affected in *RDH12*-AR mutant organoids (Figure 5d and Table S4). Thirty-five unique genes associated with RDH12 function are dysregulated. Among them, 20 are associated with visual phototransduction (18.2%, *n* = 110 genes associated with visual phototransduction), with seven downregulated and 13 upregulated; 10 associated with visual cycle in rods (10.6%, *n* = 94 genes), with seven downregulated and three upregulated; nine associated with vitamin A metabolism (21.4%, *n* = 42 genes), with four downregulated and five upregulated; eight associated with visual signal transduction in cones (34.8%, *n* = 23 genes), with five downregulated and three upregulated; 1 downregulated and one upregulated genes associated with retinol biosynthesis (15.4%, *n* = 13 genes); and one downregulated and one upregulated genes associated with retinol metabolism (5.3%, *n* = 38 genes).

**Figure 6:**
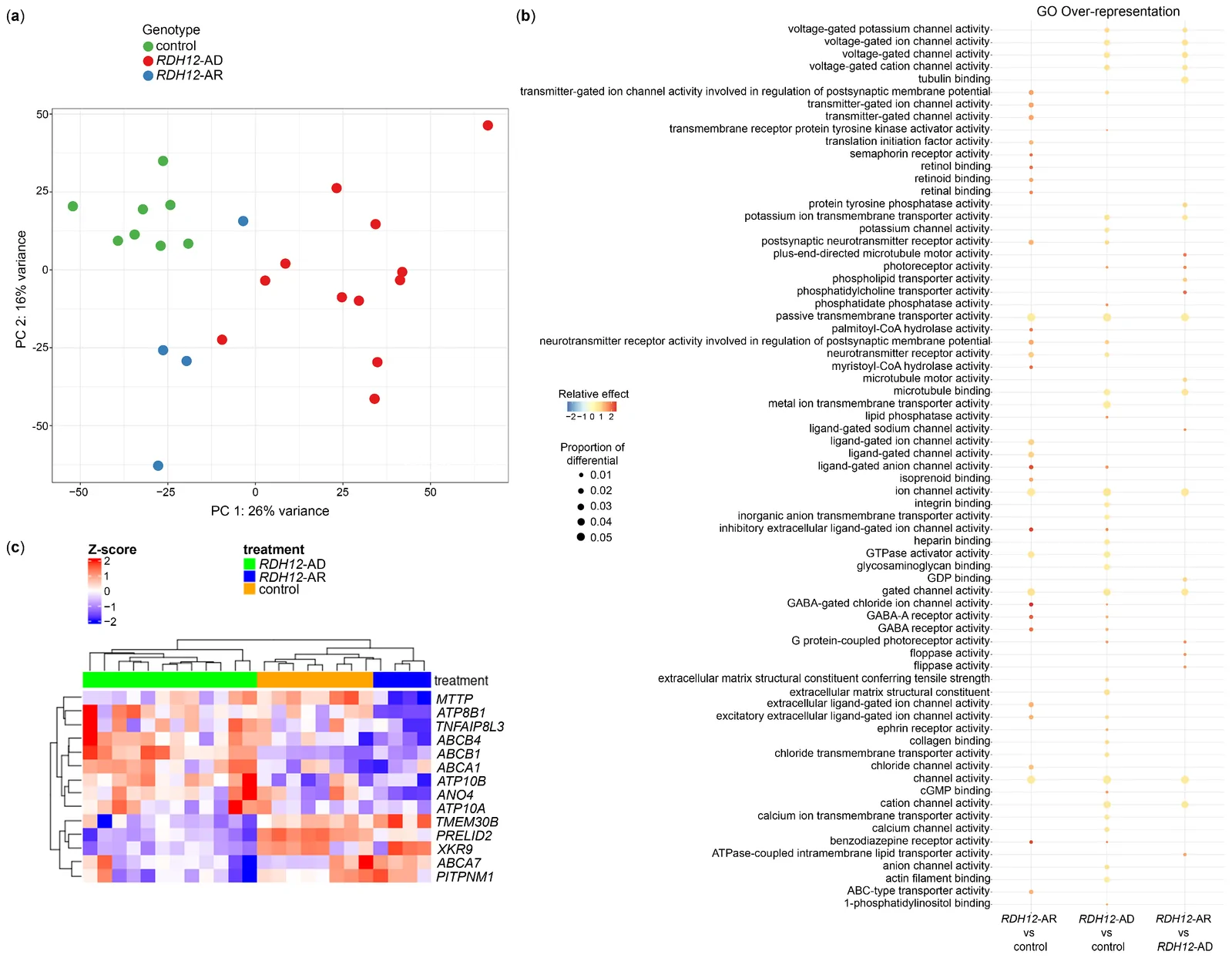
*RDH12*-AR differs transcriptionally from mature *RDH12*-AD retinal organoids. (**a**) PCA reveals three groups: unaffected control, *RDH12*-AD and *RDH12*-AR. (**b**) Gene ontology analysis shows some molecular functions are shared between *RDH12*-AD and control and *RDH12*-AR compared to control, but differ from *RDH12*-AD and *RDH12*-AR. Relative effect specifies under- (below 1) and overrepresented (above 1) pathways, supplemented with a statistically significant (hypergeometric) *p*-value. The fraction of the differentially expressed genes that belong to a specific pathway is represented by the proportion of differential genes. (**c**) Phospholipid transport was dysregulated in *RDH12*-AR compared to *RDH12*-AD retinal organoids. Standard deviation around the mean (z-score) highlights upregulated genes in red and downregulated genes in blue.

In our previous study, the transcriptomic dataset obtained from the *RDH12*-AD mature ROs model disclosed dysregulation of the vitamin A pathway and visual cycle pathway in rod and cone photoreceptors [13]. To unravel any phenotypic differences between autosomal dominant and autosomal recessive *RDH12*-retinopathies, the transcriptome obtained from both *RDH12*-AD and *RDH12*-AR mature ROs was analysed together. The comparison of *RDH12*-AD and *RDH12*-AR datasets reveals three different clusters, separated from unaffected control samples, with PC1 reaching 26% (Figure 6a). There were 1828 differentially expressed genes in *RDH12*-AR compared to *RDH12*-AD, including 1067 upregulated and 761 downregulated. GO overrepresentation showed commonalities between both *RDH12*-retinopathies (Table S5), such as passive transmembrane transporter activity, channel activity and GTPase activity (GO:0022803, GO:0015267, GO:0005216 respectively; Figure 6b and Tables S6 and S7). However, *RDH12*-AR retinal organoids overrepresented some GO compared to *RDH12*-AD samples, including ATPase-coupled intramembrane lipid transporter activity (including GO:0140326, GO:0140328, GO:0140327) and phospholipid transporter activity (including GO:0008525, GO:0005548) (Figure 6b,c and Table S8), revealing a dysregulation of phospholipid distribution [19]: *ATP10A* (LFC −3.606, *padj* < 2.773 × 10^−2^), *TNFAIP8L3* (LFC −2.837, *padj* < 4.116 × 10^−2^), *ABCB1* (LFC −2.342, *padj* < 5.909 × 10^−7^), *ATP8B1* (LFC −2.176, *padj* < 1.053 × 10^−6^), *ATP10B* (LFC −2.121, *padj* < 4.363 × 10^−2^), *ABCB4* (LFC −2.036, *padj* < 1.107 × 10^−4^), *ANO4* (LFC −1.859, *padj* < 2.573 × 10^−5^), *ABCA1* (LFC −1.072, *padj* < 4.383 × 10^−3^), *MTTP* (LFC −0.742, *padj* < 1.329 × 10^−2^), *ABCA7* (LFC 0.785, *padj* < 2.07 × 10^−2^), *PRELID2* (LFC 0.890, *padj* < 3.864 × 10^−4^), *PITPNM1* (LFC 0.896, *padj* < 2.781 × 10^−2^), *TMEM30B* (LFC 1.858, *padj* < 3.441 × 10^−2^), *XKR9* (LFC 2.284, *padj* < 3.219 × 10^−5^).

## 3. Discussion

This study reports the first human retinal organoid model of autosomal recessive *RDH12*-Leber congenital amaurosis, derived from a female with homozygous missense variant c.619A>G p.(Asn207Asp). *RDH12*-AR retinal organoids develop shorter photoreceptors compared to unaffected control retinal organoids. While mutant *RDH12* mRNA expression is unchanged, the variants affect RDH12 protein structure and its localisation to the developing photoreceptor outer segments in mature organoids. Transcriptomic signatures reveal dysregulation of phototransduction pathways and disruption of several genes associated with inherited eye diseases suggesting common pathways with other ocular diseases. *RDH12*-AR organoids differ from autosomal dominant *RDH12*-RP displaying phospholipid transport dysregulation.

Patients with recessive *RDH12*-related LCA display early photoreceptor dysfunction on electroretinography. In the RO models, cell viability remains constant between unaffected control and mutant *RDH12*-AR up to week 44, as observed with TUNEL assay. Hence, this dysfunction is unlikely due to cell death as there are an equivalent number of photoreceptors in mature *RDH12*-AR and control retinal organoids, and downregulation of three pro-apoptotic markers (*TP53AIP1*, *CASP3* and *MOAP1*) in *RDH12*-AR. The photoreceptors are dysfunctional in *RDH12*-AR retinal organoids: visual phototransduction genes are dysregulated and photoreceptor segments are shorter than unaffected controls. During photoreceptor segment morphogenesis in zebrafish [20] and in human [21,22], the inner segment elongates and acquires its cone or rod shape; 10 h later, outer segment maturation, elongation and evagination occur to form discs [23]. The reduced photoreceptor outer and inner segment length in *RDH12*-AR ROs may be due to a developmental delay or slow progressive shortening over time. AOSLO uses confocal waveguided imaging to count visible cone photoreceptors with intact outer segments, and a split-detector that can detect cone inner segments [24]. AOSLO revealed the average cone density was lower in *RDH12*-AR patients compared to unaffected individuals, but remained stable over 6 months in one *RDH12*-AR patient [25]. The 7-month-old *Rdh12*^−/−^ mouse shows no ONL or photoreceptor layer thinning, but at 10-months there is a slight reduction of photoreceptor outer-plus-inner segment length in the inferior retina [12]. Patients, mice and retinal organoid models show a slow reduction in photoreceptor outer and inner segments, without photoreceptor cell death. It is possible that the defective *RDH12*-AR photoreceptor outer segments are phagocytosed by RPE cells resulting in an accumulation of all-*trans*-retinal, which may affect its function and lead to RPE cell death with subsequent photoreceptor death as suggested in *ABCA4*-retinopathy [26]. Hence, further studies using a co-culture of retinal organoids and RPE may provide insights into the full impact on the visual cycle. These findings suggest that potential *RDH12* gene replacement could have a beneficial effect as photoreceptors are present albeit dysfunctional, hence RDH12 protein may restore function and structure.

*RDH12*-AR retinal organoids were homozygous for a missense variant and the *RDH12* mRNA level was unchanged compared to controls. At the protein level, immunostaining revealed the RDH12 protein was absent from the *RDH12*-AR photoreceptors. The missense variant may affect the RDH12 3D structure, thus potentially interfering with the antibody recognition. The polar and uncharged asparagine in position 207 is localised in an *α*-helix, while the mutated amino acid, aspartic acid, is negatively charged [27]. Tyrosine in position 200 is predicted from sequence similarity to be a proton acceptor site and Serine 175 interacts with retinal molecules (UniProt Q96NR8). It is possible that using novel RDH12 antibodies, which recognise smaller epitopes, would clarify whether RDH12 is completely absent from the photoreceptors or not. RDH12 is a NADPH-dependent dehydrogenase [2], and the presence of the mutated amino acid might affect its interaction with NADPH, the proton acceptor and its interaction with retinal, affecting overall RDH12 function. In HEK-293 stable lines, the overexpression of mutant *RDH12* with p.(Tyr226Cys), p.(Ala109Pro) or p.(Ser13*) variants led to absent or reduced levels of RDH12 protein, with reduced or absent retinol production [11]. Further experiments with HPLC and FLIM on these RO models would refine the concentration of retinal and retinol and thus the ability of affected photoreceptors to recycle retinoids [11,19].

Previous studies have shown that all-*trans*-retinal induces oxidative and ER stress, and cell death in mutant *RDH12* HEK-293 stable lines [11], in mouse 661W photoreceptors and in human ARPE-19 cell lines [28,29,30]. The same conditions were applied in mature organoids with high concentrations of H_2_O_2_ or all-*trans*-retinal, as previously reported [11], but neither *RDH12*-AR, *RDH12*-AD, nor control mature ROs appeared susceptible to stress. The absence of a detectable stress response in the positive control, after high concentrations of H_2_O_2_, is a limitation in this study. Hence, further experiments with a higher concentration of H_2_O_2_ or using other stress-induced drugs are needed to elicit a stress response in retinal organoids models after 24 h of treatment. While all-*trans*-retinal induces stress in HEK-293 stable lines 24 h after treatment, we cannot exclude that retinal organoids require a longer exposure period to induce cell responses after treatment with all-*trans*-retinal or with H_2_O_2_. As retinoic acid is present in the normal culture medium, to counteract the protective effect of retinoic acid against all-*trans*-retinal-induced stress [16,17], both RO models were cultivated in a fresh medium without retinoic acid 24 h prior to all-*trans*-retinal treatment, but both *RDH12*-AR, *RDH12*-AD and control ROs appeared to resist the stress-inducing treatment. It is possible that the retinal organoids required a longer wash-out period from retinoic acid to observe an all-*trans*-retinal induced stress. Moreover, HEK-293 stable lines only overexpressed *RDH12*; in retinal organoids both RDH12 and RDH8 are produced, where RDH8 can rescue all-*trans*-retinal-induced stress, even in mutant organoids, as observed in *Rdh12* and *Rdh12*/*Rdh8* mice models [31]. In the mutant *RDH12* HEK-293 stable lines all-*trans*-retinal induces upregulation of *NFE2L2* (or *NRF2*) and *SOD2* [11]. *SOD2* was significantly upregulated in untreated *RDH12*-AD ROs compared to untreated control (LFC 0.631, *padj* < 1.734 × 10^−2^), which suggests a response to oxidative stress. In *RDH12*-AD ROs, and WT RDH12 protein is expressed, which can reduce all-*trans*-retinal excess into all-*trans*-retinol. In untreated *RDH12*-AR retinal organoids compared to control ROs, *NFE2L2* (or *NRF2*) is downregulated (LFC −0.611, *padj* < 7.957 × 10^−3^), showing a sensitivity to oxidative stress. Further experiments are needed to unravel the all-*trans*-retinal resistance in the RO model system.

Patients affected with *RDH12*-AR LCA phenotype experience nystagmus and loss of central and peripheral vision in early childhood. We took the approach of investigating gene expression perturbations of known causative genes related to inherited retinal disease to provide insights into molecular disease mechanisms. Interestingly, *RDH12*-AR organoids show a downregulation of two genes associated with autosomal recessive congenital stationary night blindness (*LRIT3* and *RLBP1*) [32], this was also observed in *RDH12*-AD models [13]. Macular atrophy and cone dysfunction are observed in most *RDH12*-LCA patients [15], which could be justified by downregulation of cone markers; *OPN1LW*, *CNGB3*, *GNGT2*, *GRK7*, *PDE6H* and *PDE6C* [18]. Several *RDH12*-patients share shortened foveal cone outer segments, disrupted ellipsoid zone, midperipheral pigmentary changes and macular atrophy [25,33,34] similar to features seen in patients with *OPN1LW*, *CNGB3*, *PDE6H* and *PDE6C*-related cone diseases.

In *RDH12*-patients, 11-*cis*-retinal recycling is altered and blocks phototransduction, modifying ion homeostasis, and subsequently the photoreceptors release glutamate and consequently disrupt retinal electrical activity. This hypothesis is supported in both *RDH12*-AD and *RDH12*-AR models with dysregulation of genes encoding ion channels (GO:0022803, GO:0015267; *TRPM6*, *ANO3*, *CNGB3*, *CHRNA3*, *KCNIP2*, *GABRG2*, *CLCN3*, *GLRB*, *KCNC4*, *CACNB3*, *GRID1*, *GABRE*, *GABRA3*) in mature ROs [13]. Genes associated with IRDs, like *CNGB3* and *CLCN3*, cause ERG changes [35,36]. Ion concentrations challenge membrane polarisation in both photoreceptors and bipolar cells, when imbalanced, they can affect retinal cell function, providing a possible explanation for the absent or dysfunctional ERGs observed in recessive and dominant *RDH12* patients [9,15]. Pregabalin could be a good candidate for a therapeutic approach; this retinal scavenger blocks voltage-gated Ca^2+^ channels and subsequently prevents glutamate release [37]. Pregabalin reduces glutamate concentration in rats with diabetic retinopathy, reducing cell death and rescuing the retinal phenotype [37]. Recently, pregabalin was reported to reduce ER stress in in vitro HEK-293 cells overexpressing mutant RDH12 [11]; pretreatment with this drug protects against the light-induced retinal degeneration in the *Rdh12*^−/−^ mouse model [38]. Pregabalin drug testing on ROs models derived from *RDH12* patients at different doses of pregabalin may confirm this hypothesis.

**Figure 7:**
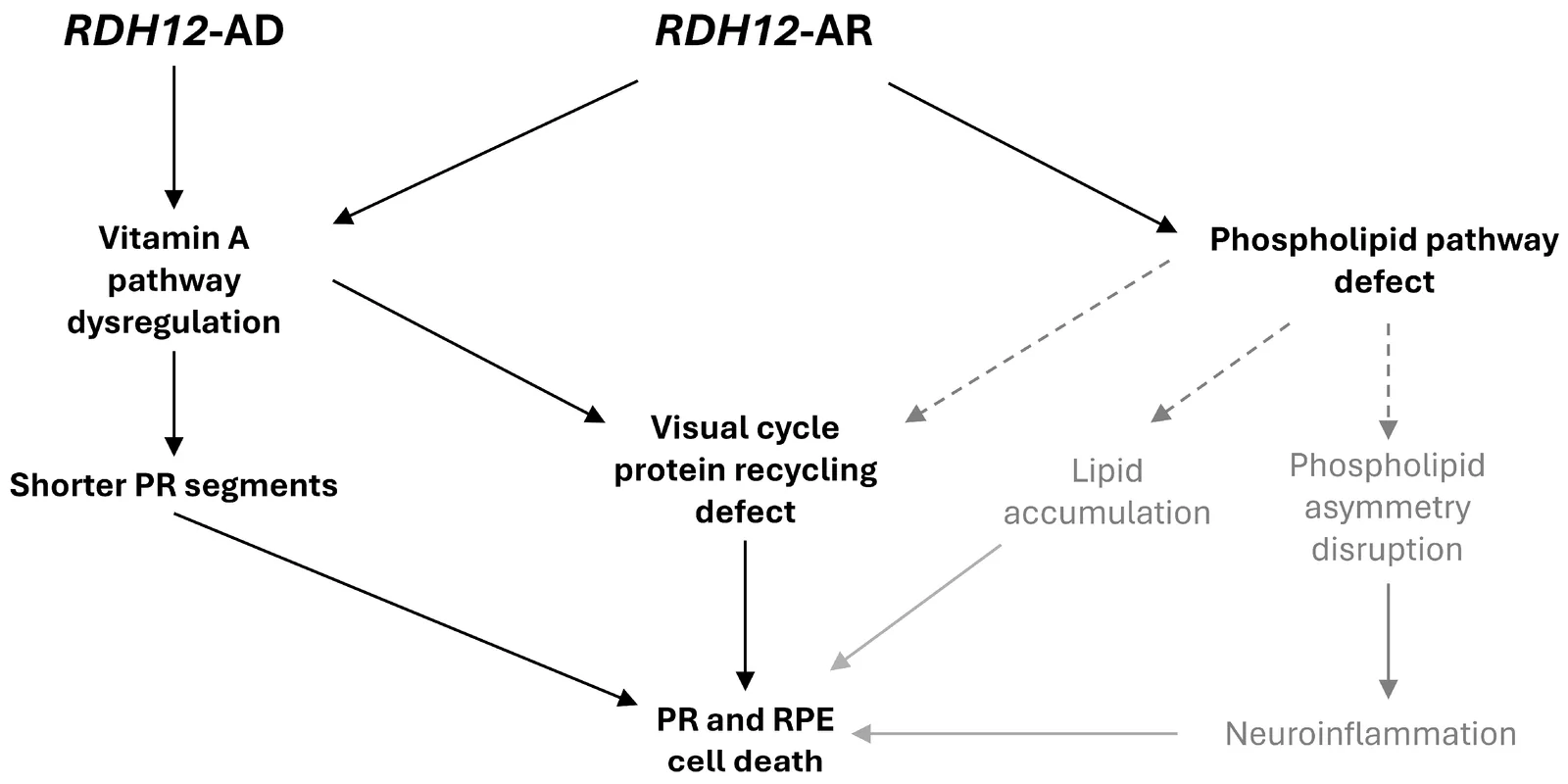
Hypothesis of pathomechanisms associated with *RDH12*-AD and *RDH12*-AR retinopathies based on transcriptomic results from mature retinal organoid models. *RDH12*-AD retinal organoids display vitamin A pathway dysregulation, which may contribute to visual cycle protein recycling defects that could lead to shortening of photoreceptor segments resulting in photoreceptor cell death. In *RDH12*-AR retinal organoids, the vitamin A pathway is also dysregulated, but phospholipid pathways are disrupted potentially also contributing to visual cycle protein recycling defects, lipid accumulation and phospholipid asymmetry disruption, which may generate neuroinflammation causing increased RPE and photoreceptor cell death. Together, these pathways may contribute to a more severe clinical phenotype in *RDH12*-AR compared to *RDH12*-AD. The mechanisms downstream of phospholipid pathway dysfunction are speculative (grey dashed arrows), based on previous neurodegenerative models described in the literature [45,46,47,49,50].

Phospholipids highly compose rod photoreceptor outer segment discs, where Rhodopsin proteins are located [39]. Lipid composition defects such as accumulation of bis(monoacylglycero)phosphate lipids, bisretinoid and lipofuscin, or reduced levels of very long-chain polyunsaturated fatty acids have been reported in patients with Stargardt disease, due to *ABCA4* or *ELOVL4* variants, respectively [40]. Moreover, the *Rdh12*^−/−^ mouse model with an *Rpe65* mutant background (Leu450) exhibits an accumulation of fluorescent *N*-retinylidene-*N*-retinylethanolamine (A2E), the major lipofuscin component in the retina [41]. Previous studies in cell lines have reported RDH12 detoxifies medium chain aldehydes, obtained from lipid peroxidation [42,43]. There are a group of lipid transporters including flippases and floppases, which maintain phospholipid asymmetry, and scramblases that disrupt phospholipid asymmetry. Both control plasma and organelle membrane constituents and subsequent subcellular protein localisation, function and turnover [3,19,40,44]. In *RDH12*-AR retinal organoids compared to *RDH12*-AD, flippases (*ATP10A*, *ATP8B1*, *ATP10B*) and floppases (*ABCA1*, *ABCB1*) are downregulated while floppase (*ABCA7*) and scramblase (*XKR9*) are upregulated, suggesting potential disruption of phospholipid asymmetry. In Alzheimer’s disease, *ABCA1* and *ABCB1* are downregulated, leading to an increase in the cholesterol lipid raft, facilitating Ab formation [45]. Moreover, downregulation of *Abca1* in the mouse model leads to age-related macular degeneration (AMD), due to accumulation of cholesterol-rich deposits resembling to subretinal drusen observed in patients [45,46]. Impairment of lipid distribution in the RPE and in photoreceptor segments might alter the recycling of visual cycle proteins and retinoids, and thus impair the phototransduction pathway in *RDH12*-AR. Further experiments, including a membrane asymmetry assay, functional phospholipid transport tests and lipidomic analysis on *RDH12* retinal organoids and on *RDH12*-related patients, may confirm phospholipid pathway defects. It has been reported that *ABCA7* is upregulated in microglial cells in Alzheimer’s disease and in frontotemporal dementia human brains, possibly leading to neuroinflammation and progressive neurodegeneration via ABCA7-mediated microglial phagocytosis [45,47,48]. Neuroinflammation via ABCA7-mediated microglial phagocytosis may be a possible mechanism contributing to the retinal degeneration in *RDH12*-AR patients, as observed in the *rd10* mouse model [49]. *MTTP* is downregulated in *RDH12*-AR organoids, as observed in patients with abetalipoproteinemia, who experience RP, due to a lipid composition defect, with low cholesterol and triglyceride levels and absence of low-density lipoproteins [50]. Transcriptomic data obtained from retinal organoid models suggest vitamin A pathway dysregulation in both *RDH12*-AD and *RDH12*-AR, however in *RDH12*-AR there is associated phospholipid pathway disruption, this may contribute to a more severe phenotype (Figure 7). Vitamin A pathway dysregulation may induce visual cycle protein recycling defects and shortening of photoreceptor segments, resulting in photoreceptor cell death. In *RDH12*-AR, phospholipid pathway disruption together with vitamin A pathway dysregulation may contribute to visual cycle protein recycling defects, lipid accumulation and phospholipid asymmetry disruption, which may contribute to neuroinflammation, and subsequent photoreceptor and RPE cell death. *RDH12*-AR transcriptomic analysis highlights the importance of performing lipidomic analysis on *RDH12*-AR retinal organoid models and on *RDH12*-AR blood samples to unravel disease-causing mechanisms associated with early-onset retinal disease [51]. These complementary analyses will help to identify possible targets for drug treatments, that could be applicable to *RDH12*-AR patients and other neurodegenerative diseases associated with similar changes in lipid composition.

In summary, we have developed and characterised the first human-derived autosomal recessive *RDH12* retinal organoid model reporting shorter photoreceptors deficient in RDH12 protein, with cone and phospholipid transport dysfunction. These insights help to further our understanding of *RDH12* disease pathophysiology [33]. With more than 170 disease-causing variants in this gene, additional representative patient samples must be studied with the appropriate isogenic controls to add further evidence. As the RPE has a critical role in 11-*cis*-retinal recycling for the visual cycle, outer segment maintenance and renewal via outer segment phagocytosis, and clearance of fluorescent bisretinoids and lipid transport [40,52], we must develop an optimised co-culture with RPE and retinal organoids to replicate the in vivo environment more closely [53,54].

## 4. Materials and Methods

### 4.1. Clinical Details

Skin biopsy was taken from a 40-year-old Pakistani female with autosomal recessive *RDH12*-Leber congenital amaurosis (LCA), with a molecularly confirmed homozygous missense variant c.619A>G p.(Asn207Asp) [14]. The patient was diagnosed at 4 years of age, with nyctalopia and progressive loss of central and peripheral vision. Her family is consanguineous, and she has two affected siblings and three affected first cousins. Her most recent best-corrected visual acuity was perception of light in both eyes at age 40. Intraocular pressure was 10 mmHg in both eyes. Anterior segment examination revealed bilateral posterior subcapsular cataracts. Fundus examination showed *RDH12*-related retinopathy with bilateral extensive bone spicule pigmentation and macular atrophy (pseudocoloboma) (Figure S4).

### 4.2. Cell Culture

Dermal fibroblasts were reprogrammed to hiPSCs from the *RDH12*-LCA patient using previously published protocols [14]. To optimise the initiation of optic vesicle production, we developed a new protocol containing DMEM with a decreasing concentration of knock-out serum replacement (KOSR) (20% for the first 4 days (included), 15% between day 5 and day 6 (included), and 10% from day 7 until day 32), 1 mM of Nicotinamide from day 0 to day 8 (included), 0.1% penicillin-streptomycin, and 1% *N*-2 supplement from day 2 [55]. Optic cups were picked between day 21 and 32, cultured in 6-well plates with 10 ng/mL of human FGF2 for 7 days [56]. Each optic cup was selected on day 50 and cultured in a 96-well plate, with the previously reported protocol to obtain laminated retinal organoids [55,57]. Retinoic acid is present in the medium from day 70 at 1µM and from day 84 at 0.5 µM until sample collection. hiPSCs derived from *RDH12*-AD, heterozygous c.759del p.(Phe254Leufs*24), and control, both male, were differentiated into retinal organoids as previously reported [14,58].

### 4.3. Retinal Organoid Characterisation and Electron Microscopy Analysis

Stage 3 retinal organoids, characterised by hair-like structures corresponding to the inner and outer photoreceptor segments [51], were cryoembedded and characterized by immunofluorescence at two different time points (week 18 and week 44; *n* = 3 organoids per line, from 2 differentiation batches, from two clones, for each timepoint), as previously reported [13,59]. Primary and secondary antibodies are detailed in [13] and in Table S9. TUNEL assay was conducted using the ApopTag Fluorescein In situ Apoptosis Detection Kit (Millipore, Burlington, MA, USA) per the manufacturer’s instructions. CRX-positive nuclei and TUNEL-positive nuclei were counted manually on Fiji (ImageJ; build 2026-05-14), as previously described in [13]. A *t*-test or a Kruskal-Wallis test was performed with *p* ≤ 0.05 considered statistically significant using R for Statistics (https://www.r-project.org/, v4.4.3). The same unaffected retinal organoids were used for CRX-positive cells and TUNEL-positive nuclei quantification as in our previous study [13].

mRNA localisation and expression of *RDH12* (1623951-C2), *RBP4* (469781) and *RLBP1* (414221) were studied in week 44 retinal organoids (*n* = 3 organoids per line) by RNAscope, following the manufacturer’s instructions (RNAscope Multiplex Fluorescent Reagent Kit v2, Advanced Cell Diagnostics (ACD), Newark, CA, USA) and the specific organoids conditions previously reported [13]. mRNA level per cell was quantified with Fiji software, as previously reported [13], and a *t*-test was performed with *p* ≤ 0.05 considered statistically significant.

At week 41–44, retinal organoids were cultivated in medium with or without 0.5 µM retinoic acid for 48 h and stressed with 100 µM all-*trans*-retinal during 24 h (*n* = 3 per condition). A two-way ANOVA was performed with *p* ≤ 0.05 considered statistically significant using GraphPad Prism (v11.0.1).

Transmission electron microscopy was performed on two retinal organoids per line at day 258 (week 37), as previously reported [13]. For each organoid, three regions 60 µm apart were studied: each photoreceptor segment from the outer limiting membrane was counted and measured in blind analysis, as previously described [13]. Photoreceptor segment counting and measurement of control retinal organoids were used for the analysis with *RDH12*-AR samples [13]. A total of 2097 and 1535 segments were counted in control and *RDH12*-AR samples, respectively. Data can only be described from this as only two retinal organoids per group were analysed and a statistical analysis cannot be done.

### 4.4. Transcriptomic Analysis in Mature Retinal Organoids

At day 303 (week 44), eight replicates (two organoids per replicate) from three differentiations from one unaffected control clone and four replicates from two differentiations from one *RDH12* AR clone were snap-frozen. RNA was isolated, quantified and cDNA generated from pooled organoids (Qiagen, Manchester, UK). cDNA libraries were sequenced on a HiSeq 2500 with paired-end reads of 150 bp using Illumina Stranded Total RNA with Ribo-Zero Plus (Illumina, Cambridge, UK). Raw reads were quality- and adapter-trimmed using cutadapt (version 3.4) [60] before alignment. Reads were mapped, and subsequently gene-level counts were generated using RSEM 1.3.1 [61] and STAR 2.7.10a [62] against the human genome GRCh38 using annotation release 95, both from Ensembl. Normalisation of raw count data and differential expression analysis were performed with the DESeq2 package (version 1.38.3) [63] within the R programming environment (version 4.2.2) [64]. The following pairwise comparisons were performed: *RDH12*-AR vs. WT; *RDH12*-AD vs. WT; and *RDH12*-AR vs. *RDH12*-AD with the contrast function, from which genes differentially expressed (adjusted *p* value being less than 0.05) between different conditions were determined. Gene lists were used to look for pathways and molecular functions with over-representation analysis using Reactome [65] and Gene Ontology (GO) [66]. Genes involved in RDH12 pathways were obtained from the PathCards database (https://pathcards.genecards.org/, accessed 29 September 2023). Unaffected control samples were the same samples used in [13]. The dataset reported in [13] was used to perform the analysis to compare *RDH12*-AD, *RDH12*-AR and unaffected control samples together (GSE283720).

## Data Availability

The bulk RNA-seq data produced in this study are available in the NCBI Gene Expression Omnibus under the access code GSE283720, including unprocessed FASTQ files and associated gene count matrices.

## Supplementary Materials

The following supporting information can be downloaded at https://www.mdpi.com/article/10.3390/ijms27198880/s1.
