# Supplementary material for: Autosomal Recessive RDH12-Leber Congenital Amaurosis Retinal Organoids Suggest Phospholipid Pathway Dysregulation
Source: Int J Mol Sci. 2026 Oct 6;27(19):8880. doi: 10.3390/ijms27198880 (PMC13665329; doi:10.3390/ijms27198880)
Supplement: Supplementary file 1 [file ijms-27-08880-s001.zip › Supplementary_Material_RDH12-AR_IJMS_PR.pdf]

Supplementary Material

Supplementary Table S1. Gene ontology (GO) over-represented in *RDH12*-AR compared to WT retinal organoids.

Supplementary Table S2. Retinal cell markers and stress-apoptosis genes are differentially expressed in mature *RDH12*-AR compared to WT retinal organoids. * mean significantly differentially expressed.

Supplementary Table S3. Thirty-six genes associated with retinal dystrophies are significantly differentially expressed in mature RDH12-AR compared to WT retinal organoids. Genes highlight in grey are reported in two or more PanelApp subgroups. * mean significantly differentially expressed.

Table S4. Thirty-five genes involved in RDH12 pathways are differentially expressed in mature RDH12-AR compared to WT retinal organoids. Genes highlight in grey are reported in two or more pathways. * mean significantly differentially expressed.

Table S5. Gene ontology (GO) over-represented in *RDH12*-AR compared to WT retinal organoids, in *RDH12*-AD compared to WT retinal organoids and in *RDH12*-AR compared to *RDH12*-AD retinal organoids.

Table S6. Genes reported in GO:0022803 passive transmembrane transporter activity and in GO:0015267 channel activity are dysregulated in *RDH12*-AD and *RDH12*-AR samples compared to WT. Genes in grey were found dysregulated in both *RDH12* mutants.

Table S7. Genes reported in GO:0005096 GTPase activator activity are dysregulated in *RDH12*-AD and *RDH12*-AR samples compared to WT. Genes in grey were found dysregulated in both *RDH12* mutants.

Table S8. Fourteen genes involved in phospholipid transporter activity are dysregulated in *RDH12*-AR retinal organoids compared to *RDH12*-AD samples.

Table S9. Primary and secondary antibodies.

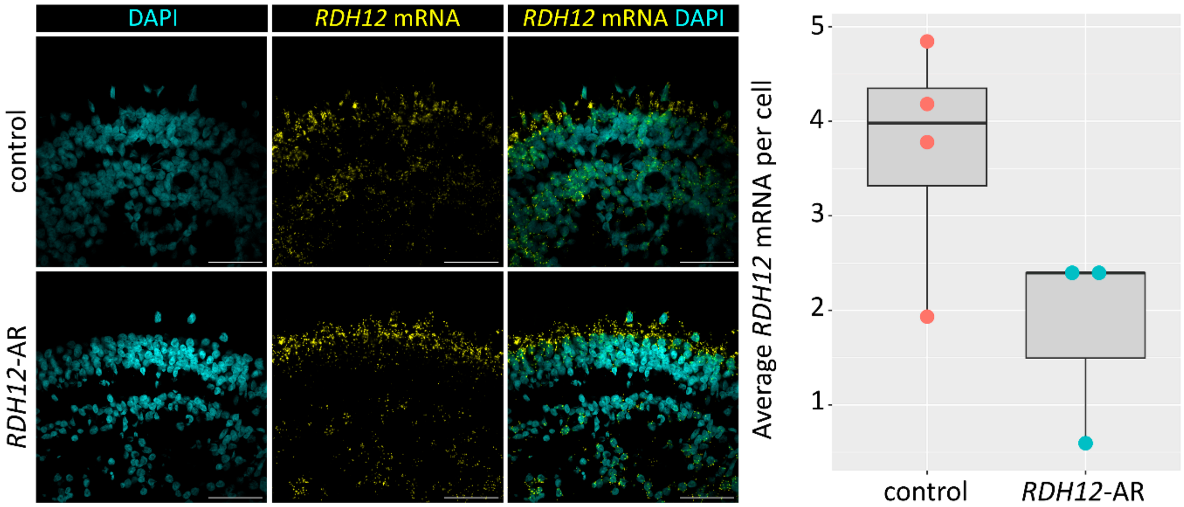

Figure S1. *RDH12* mRNA level remained similar in both control and in *RDH12*-AR retinal organoids at week 44. *RDH12* mRNA were localised at photoreceptor inner segments in both control and mutant retinal organoids, at the similar expression level (t-test, $p = 0.083$). $n = 3\text{-}4$ per line. Scale bar: 50 µm.

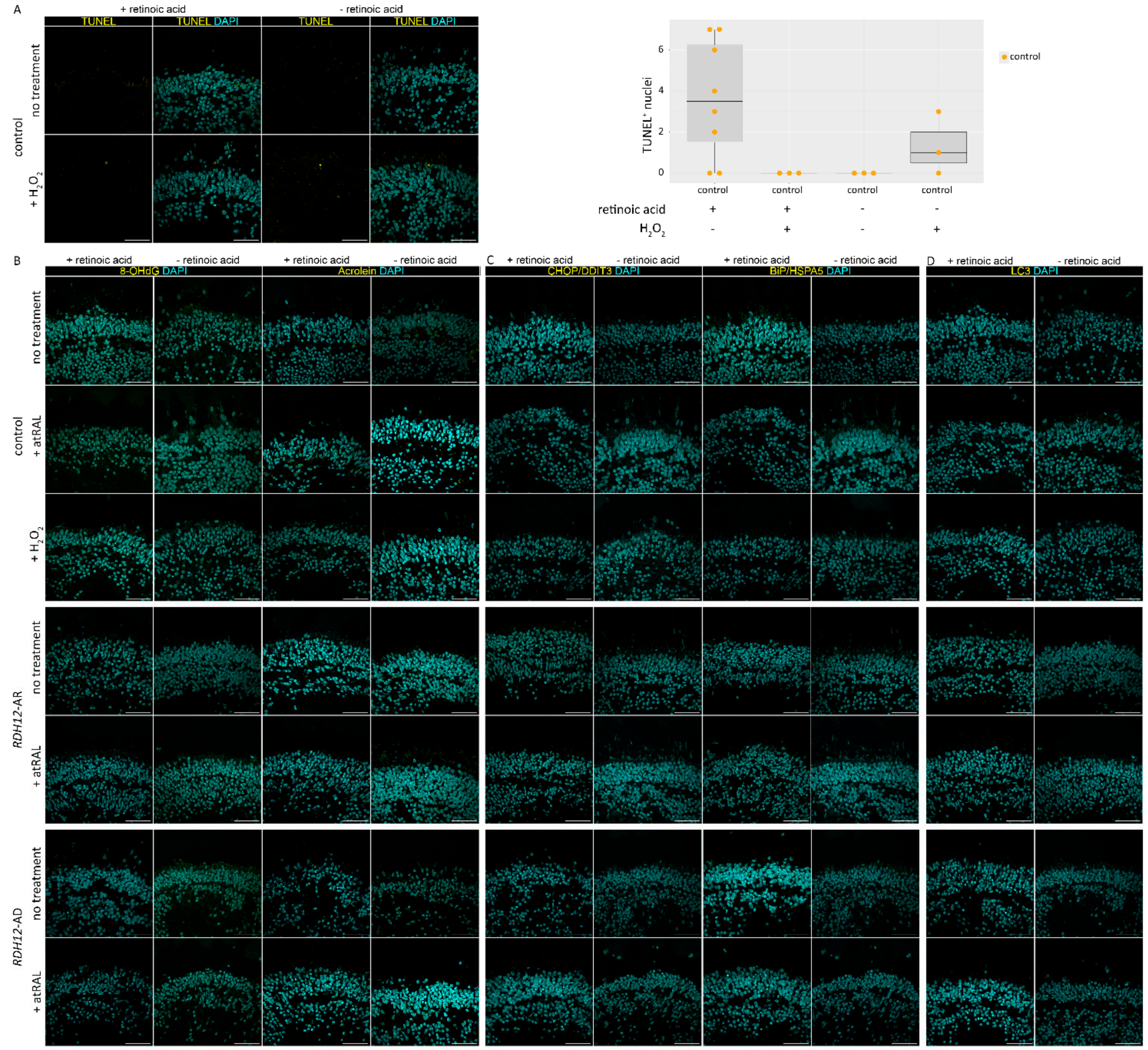

Figure S2. Control, *RDH12*-AR and *RDH12*-AD mature retinal organoids at week 41-44 are resistant to stress induced by all-*trans*-retinal (atRAL) or hydrogen peroxide (H_2O_2). (A) Control retinal organoids are resistant to H2O2-induced cell death, with or without retinoic acid in the medium (TUNEL assay, $p > 0.86$). (B) Oxidative was studied with 8-OHdG and acrolein staining; (C) ER stress with CHOP/DDIT3 and BiP/HSPA5 staining; and (D) autophagy was studied with LC3 staining, in both control, *RDH12*-AR and *RDH12*-AD mature retinal organoids. Scale bar: 50 µm.

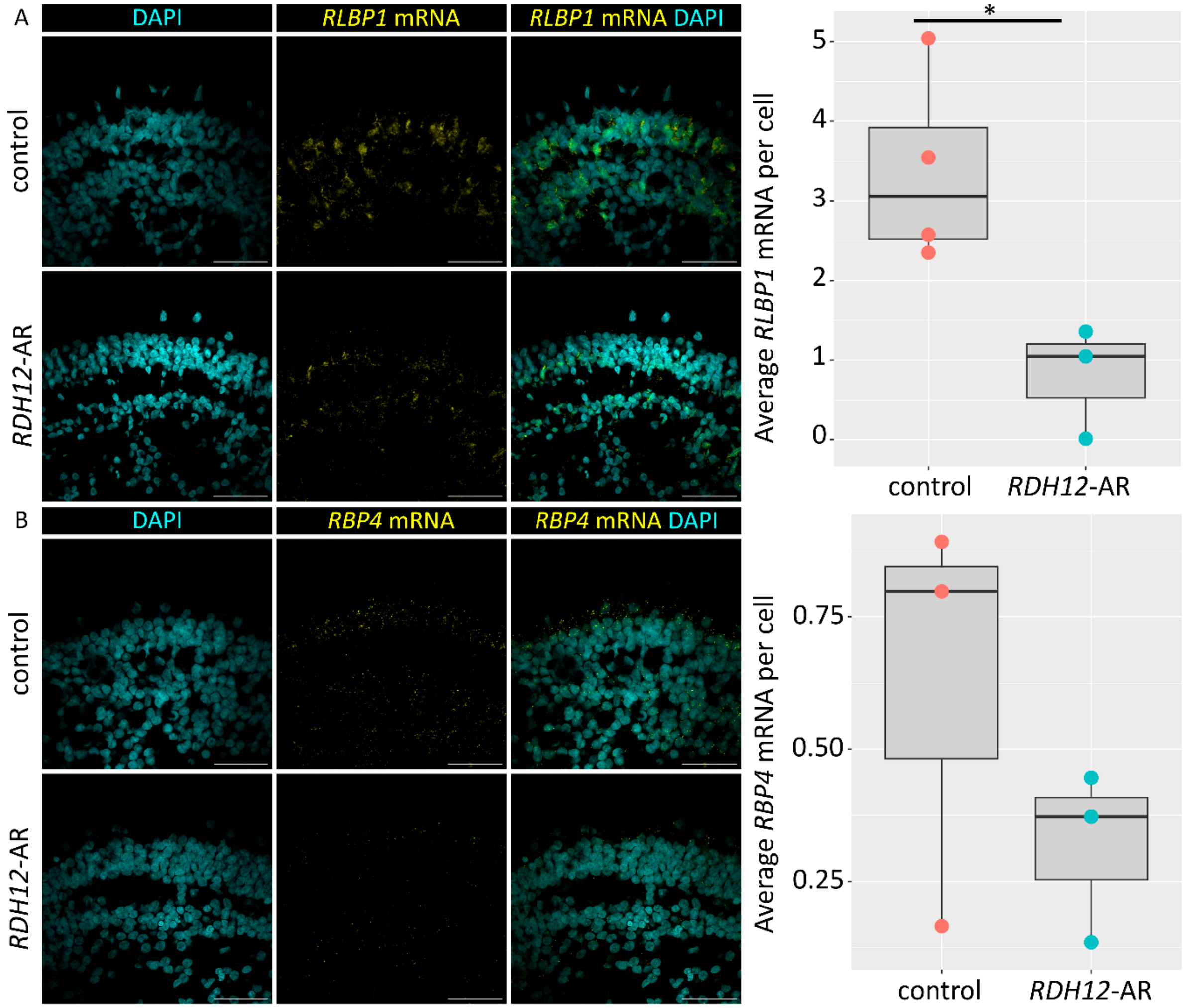

Figure S3. RNA *in situ* hybridization confirms downregulation of *RLBP1* in *RDH12*-AR mature retinal organoids at week 44. (A) *RLBP1* mRNA were localised in both outer and inner nuclear layer in both control and mutant retinal organoids. *RLBP1* expression was significantly reduced in *RDH12*-AR retinal organoids (t-test, $p = 0.018$). (B) *RBP4* mRNA was expressed in photoreceptor cells in both control and mutant retinal organoids at week 44. *RBP4* expression showed a trend toward a reduction in *RDH12*-AR retinal organoids (t-test, $p = 0.32$). $n = 3 – 4$ per line. Scale bar: 50 µm.

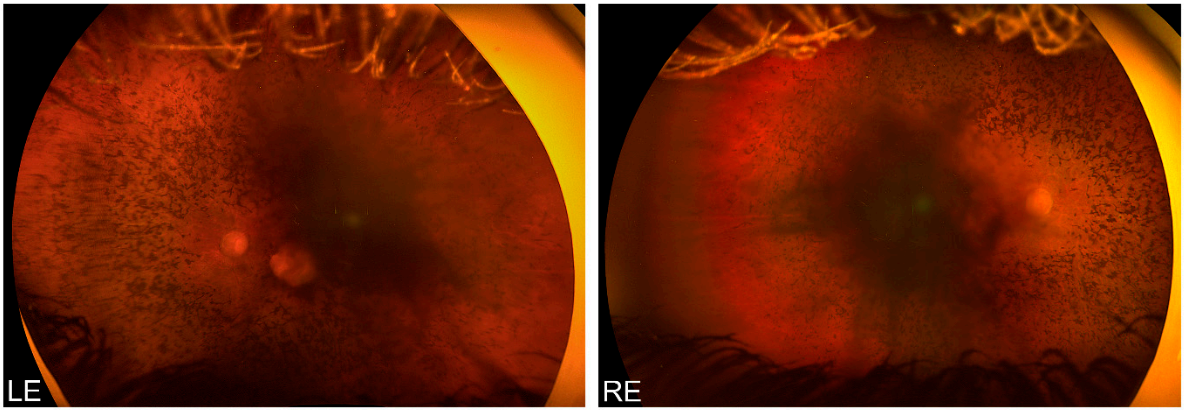

Figure S4. Fundus examination showed bilateral extensive pigment deposition in the mid periphery and macular atrophy (pseudocoloboma) in a 49 year-old woman, homozygous for the missense variant, c.619A>G p.(Asn207Asp), in *RDH12*.
